# Machine learning *vs* human experts: sacroiliitis analysis from the RAPID-axSpA and C-OPTIMISE phase 3 axSpA trials

**DOI:** 10.1093/rap/rkae118

**Published:** 2025-04-18

**Authors:** Fabian Proft, Janis L Vahldiek, Joeri Nicolaes, Rachel Tham, Bengt Hoepken, Baran Ufuktepe, Denis Poddubnyy, Keno K Bressem

**Affiliations:** Department of Gastroenterology, Infectious Diseases and Rheumatology, Charité – Universitätsmedizin Berlin, corporate member of Freie Universität Berlin and Humboldt-Universität zu Berlin, Berlin, Germany; Department of Radiology, Universitätsmedizin Berlin, corporate member of Freie Charité – Universität Berlin and Humboldt-Universität zu Berlin, Berlin, Germany; UCB Pharma, Brussels, Belgium; Department of Electrical Engineering, Center for Processing Speech and Images, KU Leuven, Leuven, Belgium; UCB Pharma, Slough, UK; Veramed, London, UK; UCB Pharma, Monheim am Rhein, Germany; UCB Pharma, Istanbul, Turkey; Department of Gastroenterology, Infectious Diseases and Rheumatology, Charité – Universitätsmedizin Berlin, corporate member of Freie Universität Berlin and Humboldt-Universität zu Berlin, Berlin, Germany; Department of Epidemiology, German Rheumatism Research Centre, Berlin, Germany; Department of Gastroenterology, Infectious Diseases and Rheumatology, Charité – Universitätsmedizin Berlin, corporate member of Freie Universität Berlin and Humboldt-Universität zu Berlin, Berlin, Germany; Department of Radiology and Nuclear Medicine, German Heart Center Munich, Munich, Germany

**Keywords:** deep learning, artificial intelligence, machine learning, axial spondyloarthritis, non-radiographic axial spondyloarthritis, radiographic axial spondylarthritis

## Abstract

**Objective:**

Diagnosis of axial spondyloarthritis (axSpA) is primarily established through the identification of the presence or absence of radiographic sacroiliitis. However, the reliability of conventional radiographs (X-rays) is undermined by significant interreader variability. A machine learning tool could reduce diagnosis time, thereby minimising interreader variability. The present study aimed to evaluate the performance of a deep learning model for detecting radiographic sacroiliitis in axSpA patients from the RAPID-axSpA (NCT01087762) and C-OPTIMISE (NCT02505542) trials.

**Methods:**

Radiographs from the RAPID-axSpA and C-OPTIMISE cohorts were retrospectively used. The deep learning model was previously trained by using a transfer learning approach on non-medical data. The model’s agreement with expert readers was tested on baseline X-rays using central reader data. Sensitivity, specificity, Cohen’s κ, positive and negative predictive values and the area under the receiver operating characteristics curve were calculated.

**Results:**

The model’s performance was evaluated in the RAPID-axSpA (*n* = 277) and C-OPTIMISE (*n* = 739) cohorts. In RAPID-axSpA, the model achieved 82% sensitivity, 81% specificity and a Cohen’s κ of 0.61, closely matching central reader performance. In C-OPTIMISE, the model demonstrated 90% sensitivity, 56% specificity and a Cohen’s κ of 0.48. The agreement between the model and central readers was 82% (RAPID-axSpA) and 75% (C-OPTIMISE).

**Conclusions:**

The tested deep learning model exhibited accurate radiographic sacroiliitis detection in axSpA patients from diverse clinical trials. The proposed deep learning model could expedite diagnosis, reduce healthcare resource usage and improve patient care pathways.

Key messages

- In our analysis, the deep learning model detects radiographic sacroiliitis in axSpA with high agreement with expert assessments, enhancing diagnostic reliability.
- Implementation of our model could streamline axSpA diagnosis, reduce healthcare resource usage and improve patient care pathways.
- The model processes images in an average of 3.5 seconds, combining efficiency with accuracy comparable to human experts, promising for widespread clinical adoption.

## Introduction

Axial spondyloarthritis [axSpA; non-radiographic (nr-axSpA) and radiographic (r-axSpA) or AS] is a chronic inflammatory rheumatic disease that primarily affects the axial skeleton, SI joints and spine [1]. Currently, imaging techniques such as conventional radiographs (X-rays) and MRI are used to aid in the diagnosis of axSpA and visualise structural damage and axial inflammation, as well as for classification [2, 3].

Radiography is often the first imaging method used for diagnosing sacroiliitis. In general, the modified New York (mNY) classification criteria is a well-established method for evaluating radiographic sacroiliitis. The imaging criterion of the mNY classification criteria for AS requires the presence of definite radiographic sacroiliitis, which is defined as sacroiliitis of at least grade 2 bilaterally or at least grade 3 unilaterally. Previous studies reported that ≈10–40% of patients with nr-axSpA progress to r-axSpA within a period of 2–10 years [4, 5]. However, radiographs remain the first recommended imaging approach when axSpA is suspected, owing to their accessibility and ease of use [6, 7].

Despite the standardized mNY classification criteria approach, X-rays have high interreader variability, and the reliability of radiographic sacroiliitis assessment remains poor regardless of whether it is performed by expert or untrained local readers. Expert central reading for clinical trial classification is time-consuming and has considerable interreader variability [8–12]. Furthermore, erroneous X-ray scores can increase the likelihood of redundant imaging, potentially delaying the time to diagnosis due to additional testing or referrals. Consequently, improving the reliability of X-ray evaluations is bound to result in a better diagnostic pathway for patients, including reduced use of healthcare resources. Currently there is a greater need for the development of sensitive and specific diagnostic algorithms, as well as more reliable imaging technologies, for the early diagnosis and progression evaluation of axSpA. The development of a radiograph analysis model based on artificial intelligence (AI) is a method that could facilitate the reliable detection of radiographic sacroiliitis.

To detect radiographic sacroiliitis with consistent repeatability, a deep learning–based model for radiograph analysis could be a promising option to ensure high levels of agreement with experts, even in non-specialized clinics [13]. A previous study on 2170 adult patients diagnosed with axSpA reported the development of a deep neural network, which was validated with expert-level performance using centrally scored images from two observational cohort studies, namely Patients With Axial Spondyloarthritis: Multicountry Registry of Clinical Characteristics and German Spondyloarthritis Inception Cohort (GESPIC) [13]. In a subsequent study, this machine learning (ML) algorithm confirmed its good performance in diagnostic settings, including patients with and without axSpA [14]. Therefore, the present study aimed to further evaluate the generalizability of a previously trained AI network [14] in r-axSpA and nr-axSpA patients from the RAPID-axSpA (NCT01087762) and C-OPTIMISE (NCT02505542) trials, which had been previously evaluated using radiographs by international expert readers not involved in the algorithm development.

## Methods

### Cohort description

In the present research, imaging data were retrospectively obtained from two previous study cohorts of patients from RAPID-axSpA and C-OPTIMISE. The RAPID-axSpA study evaluated the efficacy and safety of certolizumab pegol (CZP) in active axSpA patients without any limitation of symptom duration, while the C-OPTIMISE study assessed CZP in adult axSpA patients with a symptom duration of <5 years for sustained remission induction and maintenance. Patients in both studies were classified as r-axSpA or nr-axSpA by central independent readers/adjudicators.

### Ethical approval

As this was a post hoc analysis of anonymized data, no ethics committee or institutional review board approvals were required for this specific project. All such approvals were obtained in the original trials [15–17].

### RAPID-axSpA

RAPID-axSpA was a 204-week, phase 3, multicentre, randomized, double-blind, placebo-controlled (up to week 24), dose-blind (up to week 48) and open-label (up to week 204) study to evaluate the efficacy and safety of two dose regimens of CZP in patients with active axSpA from 83 centres across Europe, North America and Latin America. Between March 2010 and October 2011, a total of 325 patients with active axSpA were randomized 1:1:1 to placebo, CZP 200 mg every 2 weeks or CZP 400 mg every 4 weeks [15, 16].

### C-OPTIMISE

C-OPTIMISE was a two-part, multicentre, phase 3b study in adult patients with active axSpA (r-/nr-axSpA), consisting of a 48-week open-label induction period followed by a 48-week maintenance period to evaluate the induction of sustained remission and the effect of CZP maintenance dose continuation, reduction or withdrawal on flares in patients who achieved sustained remission. Patients with active adult-onset axSpA, symptom duration of <5 years and those who fulfilled the 2009 Assessment of SpondyloArthritis international Society classification criteria were included [17].

### Assessment of radiographic sacroiliitis

Using the mNY classification criteria, radiographs of the SI joints were collected, digitized, anonymised when necessary and subsequently centrally evaluated by qualified and calibrated readers [1]. RAPID-axSpA (*N* = 277) and C-OPTIMISE (*N* = 739) baseline radiographs of the SI joints were independently graded by two central reader specialists (board-certified rheumatologists with >10 years of experience in SpA imaging assessment). In case of disagreement on the presence of definite radiographic sacroiliitis (grade ≥2 bilaterally or grade ≥3 unilaterally) between the two blinded readers, radiographs were adjudicated by a third experienced reader (board-certified rheumatologist with >10 years of experience in SpA imaging assessment) who was blinded to the previous assessments. The final majority decision of the mNY classification criteria was used as a reference. No patients or central readers were involved in the studies used to pre-train the artificial network.

### Deep learning model development

The deep learning model was previously developed in another study, utilizing a deep transfer learning approach with a pre-trained network [13]. For the purposes of the current study, this model was directly employed without any additional training. This approach applies pre-trained models from non-medical domains, effectively reducing the required training data and improving model accuracy. A Ubuntu 18.04 workstation with Nvidia GeForce RTX 2080ti graphics cards and the Berlin Institute of Health’s high-performance computing cluster with an Nvidia Tesla A100 Ampere graphics card were used to train the model. Training utilized the fast.ai library built on PyTorch [18, 19], which pre-trained the convolutional neural network (ResNet-50) using a cyclical learning rate scheduler and an Adam optimizer with a weight decay of 1e-3. The input resolution was gradually extended from 224 × 224 to 768 × 768 using a progressive scaling approach, and the model was trained for 100 epochs for each resizing step using early stopping based on the highest receiver operating characteristics curve on the validation dataset. Learning rates were estimated using a rate finder, and cross-entropy loss with label smoothing was used. Additional training details are provided in Supplementary Data S1 (available at *Rheumatology Advances in Practice* online).

### Statistical analysis

Baseline X-ray images, central readings and patient characteristics were collected from both studies. Patients with scans where the SI joint was only partially visible and for which the central readings or baseline characteristics were missing were excluded. We compared the trial X-ray classification provided by the previously trained deep learning model to the patients’ central reading data. The model was blinded to the clinical information and central readings of the study population. A cut-off value of 0.724, previously derived during model development [13], was applied to binarize model predictions. The area under the receiver operating characteristics curve (AUROC), sensitivity, specificity, positive predictive value (PPV) and negative predictive value (NPV) of the previously trained deep learning model against the central reading data from both studies were calculated, along with their 95% confidence intervals (CIs) generated using accelerated bootstrapping (5000 iterations). We then assessed the agreement between the neural network and readers using the Cohen’s κ and the absolute agreement was used. Gradient-weighted class activation mapping (Grad-CAM) was used to generate activation maps and evaluate the presence of definitive radiographic sacroiliitis. Grad-CAMs provide visual explanations for model decisions by highlighting the key image regions influencing the model’s choice [20]. Finally, we randomly selected one example of false positive, false negative, true positive and true negative prediction from each study to evaluate the Grad-CAMs.

## Results

**Figure 1.**
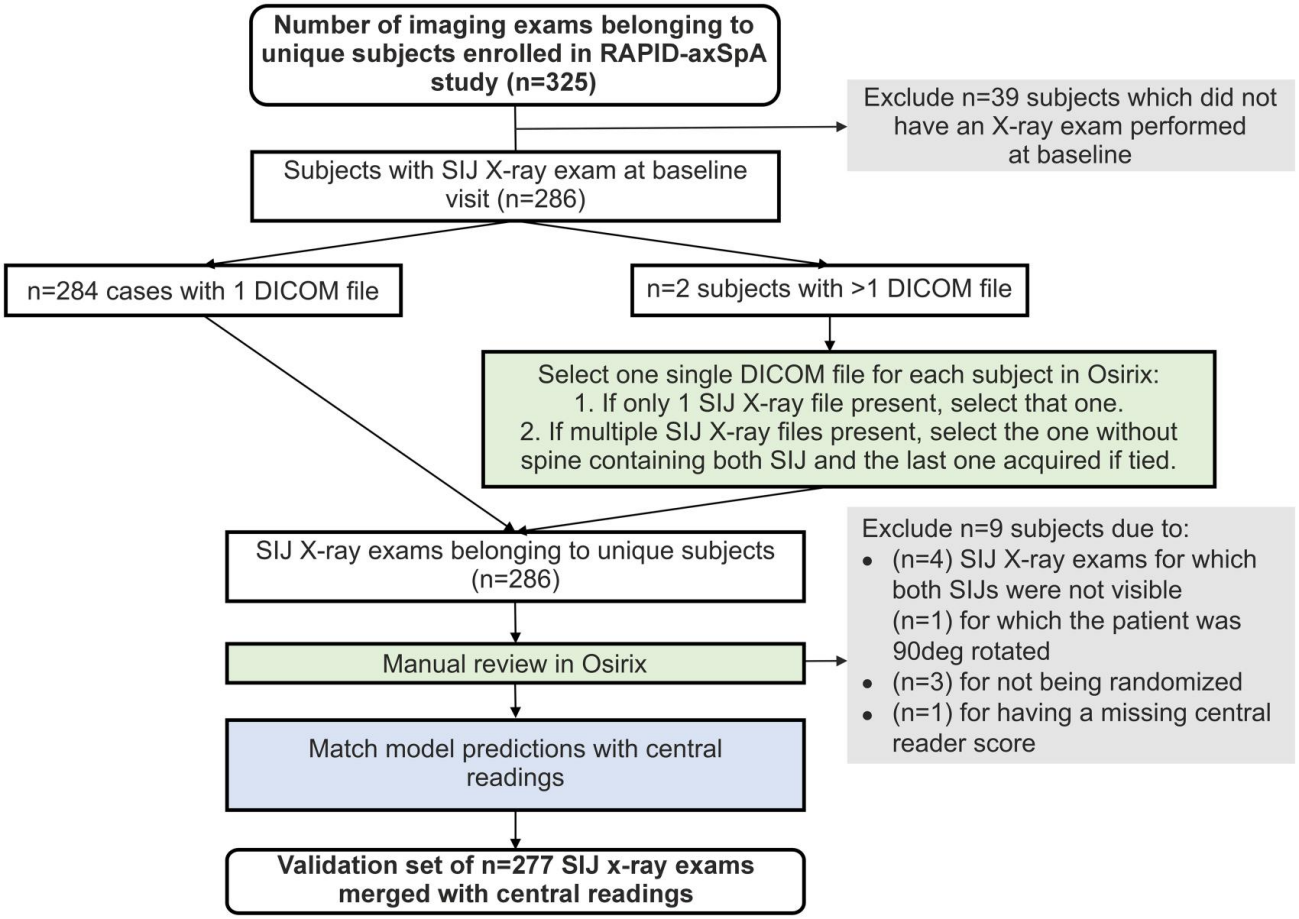
Data flow for the RAPID-axSpA phase 3 axSpA trial. The green box highlights a manual activity performed by an engineer. The blue box highlights an automated procedure matching predictions and readings on a unique patient identifier

**Figure 2.**
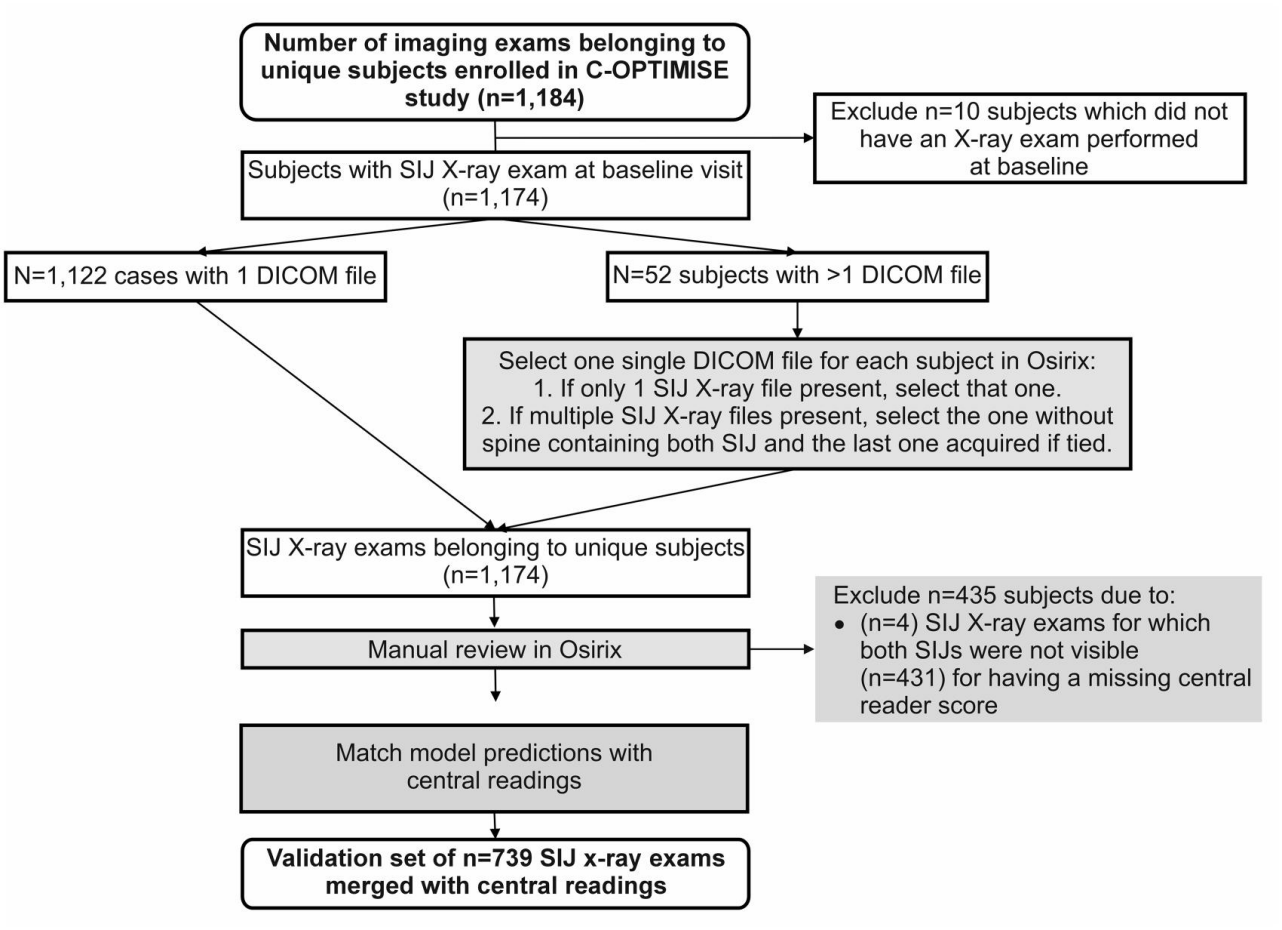
Data flow for the C-OPTIMISE phase 3 axSpA trial. The green box highlights a manual activity performed by an engineer. The blue box highlights an automated procedure matching predictions and readings on a unique patient identifier

The data flow diagrams for RAPID-axSpA and C-OPTIMISE are shown in Figs. 1 and 2, respectively. In the RAPID-axSpA study, 286 of 325 enrolled patients had SI joint X-rays at the baseline visit; a test set of 277 patients with SI joint X-rays combined with central readings was then processed by the deep learning model. The study data from the 1184 patients enrolled in C-OPTIMISE included 1174 patients with SI joint X-ray exams at the baseline visit; a test set of 739 patients with SI joint X-ray exams combined with central readings was then processed by the deep learning model.

The inference script processed the RAPID-axSpA and C-OPTIMISE images for 15 and 45 min, respectively, and a total of 1016 unique X-ray images were included in the analysis. In RAPID-axSpA, 277 patients [mean age 39.5 years (s.d. 12.1); 112 females] were investigated, while 739 patients [mean age 32.9 years (s.d. 7.0); 225 females] were investigated in C-OPTIMISE. The C-OPTIMISE trial included a symptom duration criterion of <5 years for inclusion; thus the mean symptom duration in the RAPID-axSpA study was longer (10.2 years) compared with that in the C-OPTIMISE study (3.3 years). However, the duration was comparable between subgroups within each study. Similar trends in the mean disease duration (6.3 in RAPID-axSpA *vs* 2.1 in C-OPTIMISE) were noted. The mean C-reactive protein (CRP) was slightly higher in the cohort from RAPID-axSpA than in that from C-OPTIMISE (18.6 *vs* 14.2 mg/l). The mean baseline values of the ASDAS, BASDAI, BASFI and BASMI were similar between all populations in both cohorts. The baseline characteristics of both cohorts are presented in Supplementary Table S1 (available at *Rheumatology Advances in Practice* online).

Of the 277 (r-axSpA, 181; nr-axSpA, 96) and 739 (r-axSpA, 403; nr-axSpA, 336) patients in the RAPID-axSpA and C-OPTIMISE study cohorts, the neural network detected definite radiographic sacroiliitis (classification as r-axSpA) on 166 X-rays in the RAPID-axSpA cohort and 512 X-rays in the C-OPTIMISE cohort. The confusion matrix tables are presented in Table 1.

**Table 1.** Confusion matrix table for the RAPID-axSpA and C-OPTIMISE studies

RAPID-axSpA	mNY (yes)	mNY (no)	Total
ML algorithm mNY (yes)	148	18	166
ML algorithm mNY (no)	33	78	111
Total	181	96	277
C-OPTIMISE			
ML algorithm mNY (yes)	364	148	512
ML algorithm mNY (no)	39	188	227
Total	403	336	739

**Figure 3.**
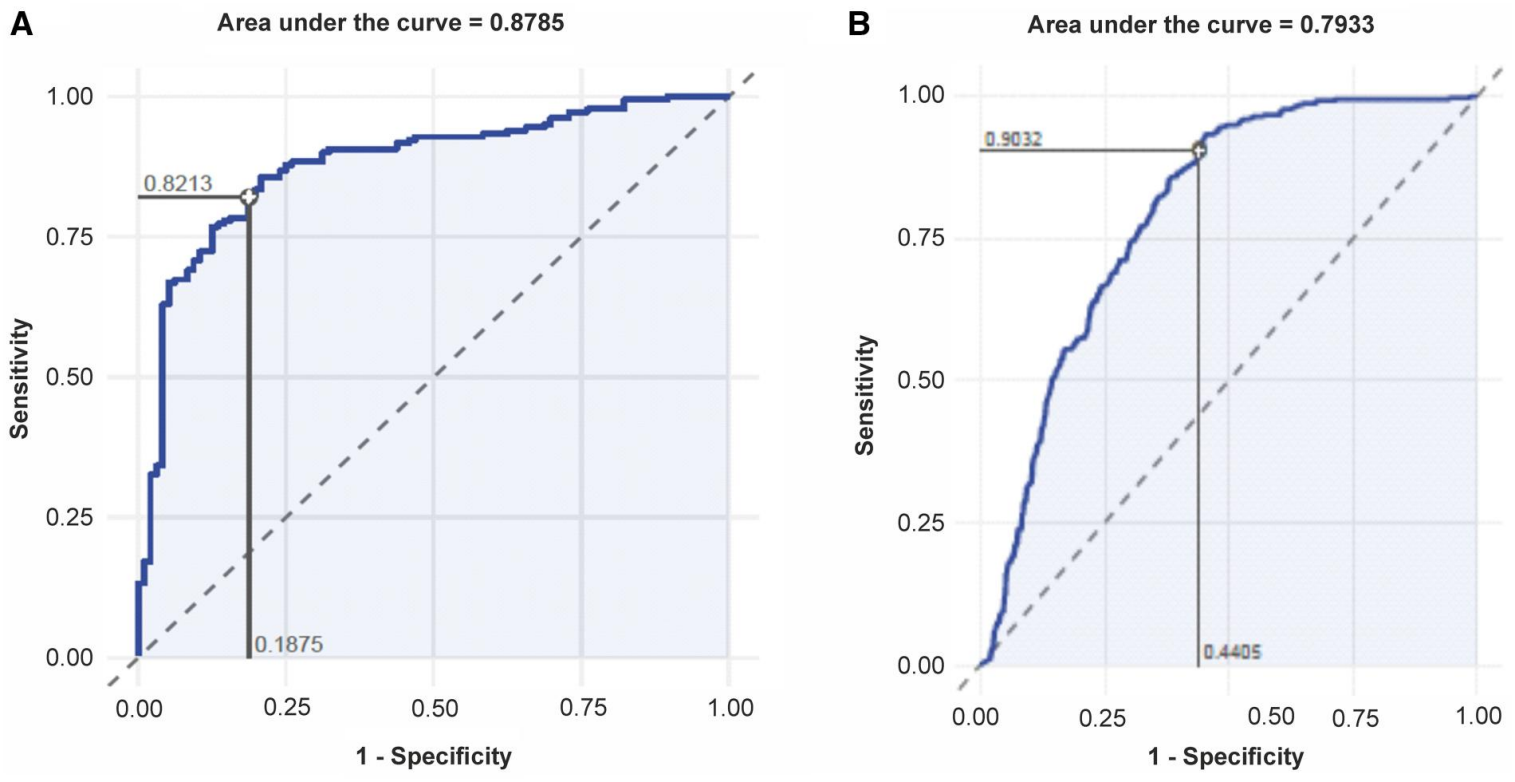
Receiver operator characteristics curve assessing the ability of the evaluated ML model to discriminate between r-axSpA and nr-axSpA outcomes: **(A)** RAPID-axSpA and **(B)** C-OPTIMISE

Compared with the central assessment in the RAPID-axSpA study, the neural network exhibited a sensitivity of 0.82 (95% CI 0.78, 0.86), specificity of 0.81 (95% CI 0.75, 0.87), Cohen’s κ of 0.61 (95% CI 0.51, 0.70) and 82% absolute agreement. The κ statistic between the central readers was 0.81 and the percent agreement was 91.2%. The PPV, NPV and AUROC were 0.89 (95% CI 0.86, 0.93), 0.70 (95% CI 0.64, 0.77) and 0.88, respectively, for the recognition of definite radiographic sacroiliitis (Table 2 and Fig. 3). There was substantial agreement between all readers, including the adjudicator. The adjudicator’s agreement was slightly more in line with the ML algorithm than that of readers 1 and 2. The consistency of sacroiliitis eligibility assessments among central readers is summarized in Supplementary Table S2 (available at *Rheumatology Advances in Practice* online).

**Table 2.** Performance results comparing the deep learning model with central readings in the RAPID-axSpA and C-OPTIMISE X-ray image cohorts

Results	RAPID-axSpA (*n* = 277)	C-OPTIMISE (*n* = 739)
Sensitivity	0.82 (95% CI 0.78, 0.86)	0.90 (95% CI 0.88, 0.93)
Specificity	0.81 (95% CI 0.75, 0.87)	0.56 (95% CI 0.52, 0.60)
Cohen’s κ	0.61 (95% CI 0.51, 0.70)	0.48 (95% CI 0.41, 0.54)
PPV	0.89 (95% CI 0.86, 0.93)	0.71 (95% CI 0.68, 0.74)
NPV	0.70 (95% CI 0.64, 0.77)	0.83 (95% CI 0.79, 0.87)
AUROC	0.88	0.79
Agreement with adjudicated score	0.82 (95% CI 0.78, 0.85)	0.75 (95% CI 0.72, 0.77)

**Figure 4.**
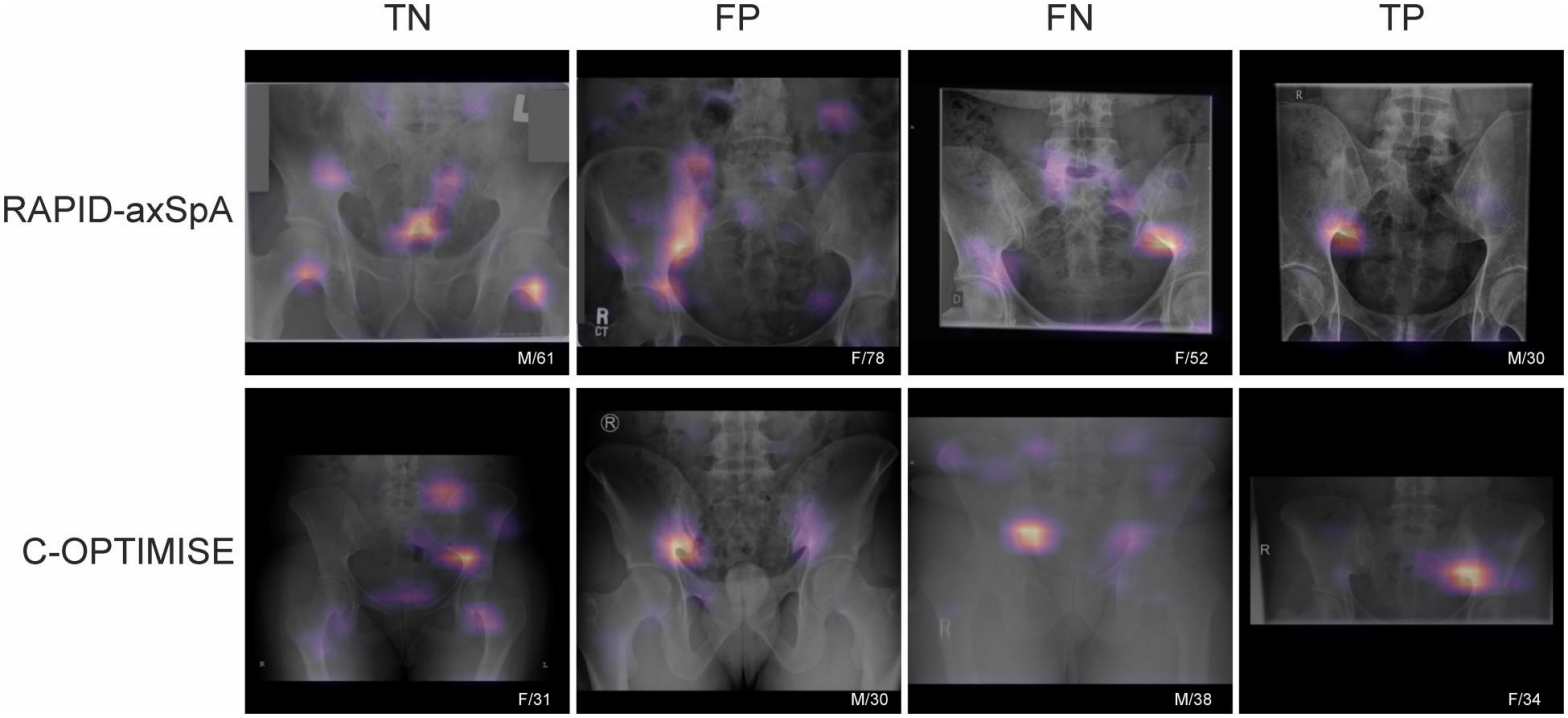
Illustration of the algorithm image analysis results on selected examples from RAPID-axSpA (first row) and C-OPTIMISE (second row). Each image contains the original X-ray overlaid by the Grad-CAM image generated by the algorithm, highlighting the image areas that had the greatest impact on the algorithm’s prediction. The colour scale of the Grad-CAM overlay images ranges from purple (low impact) to yellow (high impact). To compare the algorithm predictions with the central readings for each study, we present TN, FP, FN and TP examples in columns 1–4, respectively. The caption of each image displays the sex and age of the subject. FN: false negative; FP: false positive; TN: true negative; TP: true positive

Compared with the central assessment in the C-OPTIMISE study, the neural network achieved a sensitivity of 0.90 (95% CI 0.88, 0.93), specificity of 0.56 (95% CI 0.52, 0.60), Cohen’s κ of 0.48 (95% CI 0.41, 0.54) and 75% absolute agreement. The κ statistic between the central readers was 0.45 and the percent agreement was 73.0%. The PPV was 0.71 (95% CI 0.68, 0.74), NPV was 0.83 (95% CI 0.79, 0.87) and AUROC was 0.79 for recognition of definite radiographic sacroiliitis (Table 2 and Fig. 3). Fig. 4 depicts the original X-ray images superimposed with Grad-CAM images produced by the algorithm.

## Discussion

To our knowledge, this is the first large-scale ML study evaluating SI joint X-ray scans that had been previously evaluated and adjudicated by an independent group. In the present study, we evaluated the ability of an AI model to detect radiographic sacroiliitis on radiographs in an external dataset from two large multicentre clinical trials involving both r- and nr-axSpA patients. With this model, we demonstrated high agreement with expert readers, with an average processing time of 3.5 s that aligns closely with the performance of human experts.

In the absence of reliable laboratory biomarkers and a high prevalence of chronic back pain—the leading clinical symptom of axSpA—in the general population, imaging plays an important role in the diagnosis of axSpA. Owing to the high costs and restricted availability of MRI, X-rays have become the preferred option, thereby rendering radiographs the first recommended imaging modality used to examine patients with axSpA. Although the detection of definite radiographic sacroiliitis is vital for both the diagnosis and classification of axSpA, X-rays are not sufficiently reliable in detecting sacroiliitis due to inter- and intrareader variability [9, 21].

Furthermore, a reliable assessment of SI X-rays is required in most contemporary trials in the field of axSpA (i.e. classification of patients as r- or nr-axSpA). In the present study, we used data from patients diagnosed with axSpA collected from multicentre clinical trials to test the model. This model demonstrated high accuracy in achieving performance comparable to that of two human experts. While the percent agreement between the central blinded readers was 91.2% (RAPID-axSpA) and 73.0% (C-OPTIMISE), the agreement between the ML algorithm and central reader was 82% (RAPID-axSpA) and 75% (C-OPTIMISE). Test data from the RAPID-axSpA study showed a greater ML agreement compared with C-OPTIMISE, while the model in the C-OPTIMISE study showed less specificity. This divergence could stem from varying study criteria, which rendered it more challenging for the model to accurately distinguish between the conditions. Notably, RAPID-axSpA excluded cases with a symptom duration of <5 years, potentially leading to enhanced concordance between central readers and the model. Conversely, C-OPTIMISE, with included cases with a symptom duration of <5 years, displayed relatively lower agreement. This aspect introduces a new challenge, as the condition might not have fully manifested or progressed. Additional factors such as age, sex, disease duration, CRP levels and study time frames could contribute to the discrepancies noted in the agreement between the two cohorts. However, compared with the expert evaluations alone in the C-OPTIMISE study, the ML algorithm showed agreement on par with the experts when compared with the evaluations provided solely by the experts. This suggests that the ML algorithm showed an expected drop in performance when exposed to more challenging baseline factors for diagnosis, similar to the agreement between expert readers. The scoring agreement observed in the RAPID-axSpA dataset was on par with, or marginally lower than, the levels noted in the GESPIC [13] and OptiRef [14] datasets. Notably, the deep learning algorithm employed in this study was on par with an experienced reader skilled in radiographic sacroiliitis evaluation within the RAPID-axSpA dataset. This was also consistent with prior testing outcomes involving the GESPIC [13] and OptiRef [14] datasets, thereby establishing the high reliability and robustness of the model in the RAPID-axSpA set.

ML, particularly in the context of deep learning, may show limited applicability and suboptimal performance when exposed to new data. A meta-analysis of published data has highlighted various flaws, including a lack of comparative analysis between the performance metrics of the AI model and those of a human expert [22]. However, the use of different datasets from different studies revealed that the models could be adequately generalizable [23, 24]. In the present study, a heterogeneous test dataset was also used that included radiographs from several imaging sites, a large sample size, different and independent readers, and the comprehensive stress test on an existing model, especially from one test set, exhibited good generalizability.

By overcoming interreader variability, this model has the potential to reduce diagnosis time if used as a standard tool in practice. By integrating automated ML tools, diagnostic time could be decreased and a more optimal screening could be performed in other disease areas. In false negative cases scored by human readers, the algorithm could help identify the image as positive and might therefore overcome the necessity of using another imaging modality such as MRI, which would delay diagnosis time due to additional imaging. These approaches should be tested in the future to evaluate the impact on the time to diagnosis for r-axSpA.

Our results reveal the potential for classification purposes in multicentre axSpA trials in the future, providing a reproducible and cost-effective tool without unnecessary time delays. The model can easily be shared with various end users as a containerized application or widely used as a web service, without the need for further training or fine-tuning. It is noteworthy that despite the differences among baseline characteristics and all the uncertainty related to radiographic sacroiliitis assessment, the deep learning algorithm–based classification was balanced and demonstrated a moderate level of agreement between the algorithm and central human readers.

Our study has few limitations. First and foremost, the agreement between central readers and the algorithm was not absolute; a similar challenge is observed between readers. Unlike simple mathematical calculations and despite the fact that the scoring system is clearly defined, the input of readers is subjective, rendering the final score human dependent and therefore variable. Although our study had a fairly large sample size and a detailed imaging charter for reading standards, it was not fully representative of clinical practice. The evolution of reading and X-ray standards from the beginning of the RAPID-axSpA study until the present may have changed over time. In addition, the radiographs in both the RAPID-axSpA and C-OPTIMISE studies were scored by different experts with varying reading standards and different reading time points, thus leading to a plausible interreader variability. It is also known that image quality and standardization is a prominent issue in X-rays, especially in SI X-rays, thus the variation of X-ray optimality in multicentre studies might be considered as a limitation. Future work should investigate the impact of known reader variability on the model’s performance and investigate the performance with adequate sensitivity analyses in different subgroups according to patient and acquisition characteristics.

Our findings revealed that a pre-trained artificial neural network can allow the accurate detection of definite radiographic sacroiliitis within seconds for the diagnosis and classification of axSpA, which is comparable to expert human readings, thus providing a reproducible and cost-effective tool in future multicentre axSpA studies.

## Supplementary Material

rkae118_Supplementary_Data

## Data Availability

The datasets generated and/or analysed during the current study are available from the corresponding author upon reasonable request. The algorithm is available for research purposes from https://rad-ai.charite.de/spa.
