# Supplementary material for: Machine learning vs human experts: sacroiliitis analysis from the RAPID-axSpA and C-OPTIMISE phase 3 axSpA trials
Source: Rheumatol Adv Pract. 2025 Apr 18;9(2):rkae118. doi: 10.1093/rap/rkae118 (PMC12007599; doi:10.1093/rap/rkae118)
Supplement: rkae118_Supplementary_Data [file rkae118_supplementary_data.docx]

**SUPPLEMENTARY MATERIAL:**

**Supplementary Data S1**

As a loss function, cross-entropy label smoothing was used, which reduced high-confidence predictions of the models, thus supporting regularisation and avoiding overfitting with subsequent improved generalisation of the models on new data (e.g. test dataset). Using a learning rate range test, the optimal learning rate for training was determined^18^. Model training was performed with cyclical (Smith LN 2017), discriminative learning rates^19^ and a progressive resizing approach, starting with image sizes of 224 × 224 pixels, which is the default input size for the ImageNet pre-trained ResNet-50, and subsequently increasing the resolution to 512 × 512 pixels and then to 768 × 768 pixels. During training, only the last two classification layers of the model were initially trained, with the weights of the other network layers remaining frozen. A total of 100 epochs were trained, monitoring the area under the receiver operating characteristics curve (AUROC) on the validation dataset and saving model weights on every improvement. After 100 epochs, the weights of the model with the highest AUROC value were re-loaded, the model was unfrozen and again trained for another 100 epochs (training all layers of the network), while monitoring the AUROC and saving the weights at every improvement. This approach was repeated for all image resolutions. The size of the mini batches was 64 for 224 × 224 pixels, 32 for 512 × 512 pixels and 84 for 768 × 768 pixels. Training for lower resolutions could be performed at our local workstation, while computation for 768 × 768 pixels was conducted on the HPC for Research cluster of the Berlin Institute of Health. Overall, model training took approximately 24 h on our local machine and an additional 6 h on the cluster. After training, we employed Gradient-weighted Class Activation Mapping (Grad-CAM) to create activation maps and verify that the model used the sacroiliac joints to determine the presence of definite radiographic sacroiliitis^18^.

The previously trained model was loaded from the GitHub repository <https://github.com/jlvahldiek/docker-deploy-spa> to build and install a docker image of the trained model in the UCB data centre (JN). Next, baseline, sacroiliac joints and anterior-posterior x-ray images were loaded as Digital Imaging and Communications in Medicine (DICOM) files from the RAPID-axSpA and C-OPTIMISE study data and processed by the inference script of the docker image. The inference step produced a binary outcome ‘prediction_r-axSpA_balanced_cutoff’ after the application of the optimal cut-off value of 0.724 defined in^20^ for every x-ray image processed. A subset of the C-OPTIMISE images required additional and manual pre-processing steps that set the Photometric Interpretation DICOM tag to ‘MONOCHROME1’ to correct issues stemming from this parameter surfacing during data acquisition. All other study images included in this study were processed by the previously trained model as described in^20^.

**Supplementary Table S1: Baseline demographics and disease characteristics for patients enrolled in the RAPID-axSpA and C-OPTIMISE**

| **Baseline characteristics** | **RAPID-axSpA** | | | | | **C-OPTIMISE** | | | | |
| --- | --- | --- | --- | --- | --- | --- | --- | --- | --- | --- |
|  | **Overall** | **r-axSpA** | | **nr-axSpA** | | **Overall** | **r-axSpA** | | **nr-axSpA** | |
|  | Total (N = 277) | Study Results mNY = Yes (N = 181) | ML Algorithm mNY = Yes (N = 166) | Study Results mNY = No (N = 96) | ML Algorithm mNY = No (N = 111) | Total (N = 739) | Study Results mNY = Yes (N = 403) | ML Algorithm mNY = Yes (N = 512) | Study Results mNY = No (N = 336) | ML Algorithm mNY = No (N = 227) |
| Age, years, mean (SD) | 39.5 (12.1) | 39.2 (11.6) | 39.4  (11.9) | 40.0  (13.0) | 39.7  (12.3) | 33.0  (7.0) | 33.7  (6.8) | 33.2  (6.8) | 32.1  (7.2) | 32.6  (7.5) |
| Sex (male), n (%) | 165 (59.6) | 122 (67.4) | 115 (69.3) | 43 (44.8) | 50 (45.0) | 514 (69.6) | 317 (78.7) | 382 (74.6) | 197 (58.6) | 132 (58.1) |
| Symptom duration, years, mean (SD) | 10.23  (9.42) | 10.63  (9.55) | 11.09  (9.60) | 9.47  (9.15) | 8.94  (9.02) | 3.32  (2.23) | 3.70  (2.52) | 3.49  (2.39) | 2.86  (1.72) | 2.92  (1.77) |
| Disease duration, years, mean (SD) | 6.32  (7.00) | 7.05  (7.50) | 7.08  (7.24) | 4.96  (5.75) | 5.20  (6.51) | 2.14  (1.72) | 2.46  (1.79) | 2.23  (1.76) | 1.75  (1.55) | 1.94  (1.62) |
| HLA-B27 (+), n (%) | 210 (75.8) | 150 (82.9) | 140 (84.3) | 60 (62.5) | 70 (63.1) | 609 (82.4) | 355 (88.1) | 444 (86.7) | 254 (75.6) | 165 (72.7) |
| BMI, kg/m^2^, mean (SD) | 27.99  (5.97) | 27.90  (5.54) | 28.26  (6.01) | 28.18  (6.75) | 27.59  (5.91) | 25.71  (4.87) | 25.65  (4.67) | 25.74  (4.70) | 25.78  (5.11) | 25.62  (5.25) |
| BMI class (kg/m^2^), n (%) | | | | | | | | | | |
| <18.5 | 6 (2.2) | 2 (1.1) | 3 (1.8) | 4 (4.2) | 3 (2.7) | 29 (3.9) | 17 (4.2) | 18 (3.5) | 12 (3.6) | 11 (4.8) |
| 18.5–<25 | 89 (32.1) | 56 (30.9) | 46 (27.7) | 33 (34.4) | 43 (38.7) | 323 (43.7) | 163 (40.4) | 212 (41.4) | 160 (47.6) | 111 (48.9) |
| 25–<30 | 89 (32.1) | 67 (37.0) | 63 (38.0) | 22 (22.9) | 26 (23.4) | 248 (33.6) | 152 (37.7) | 190 (37.1) | 96 (28.6) | 58 (25.6) |
| ≥30 | 88 (31.8) | 54 (29.8) | 52 (31.3) | 34 (35.4) | 36 (32.4) | 130 (17.6) | 71 (17.6) | 88 (17.2) | 59 (17.6) | 42 (18.5) |
| Missing | 5 (1.8) | 2 (1.1) | 2 (1.2) | 3 (3.1) | 3 (2.7) | 9 (1.2) | 0 (0.0) | 4 (0.8) | 9 (2.7) | 5 (2.2) |
| CRP, mg/L, mean (SD) | 18.57  (22.99) | 20.53  (24.32) | 21.90  (25.36) | 14.87  (19.84) | 13.60  (17.88) | 14.21  (18.39) | 16.47  (18.69) | 15.73  (18.61) | 11.43  (17.65) | 10.76  (17.44) |
| CRP category, mg/L, n (%) <stratified as ≤ULN or >ULN for C-OPTIMISE study > | | | | | | | | | | |
| ≤15 mg/L | 163 (58.8) | 101 (55.8) | 89 (53.6) | 62 (64.6) | 74 (66.7) | 409 (55.3) | 192 (47.6) | 260 (50.8) | 217 (64.6) | 149 (65.6) |
| >15 mg/L | 114 (41.2) | 80 (44.2) | 77 (46.4) | 34 (35.4) | 37 (33.3) | 321 (43.4) | 211 (52.4) | 248 (48.4) | 110 (32.7) | 73 (32.2) |
| ASDAS, mean (SD) | 3.88  (0.87) | 3.99  (0.88) | 4.03  (0.90) | 3.68  (0.83) | 3.66  (0.79) | 3.71  (0.82) | 3.84  (0.82) | 3.79  (0.82) | 3.56  (0.79) | 3.53  (0.79) |
| BASDAI, mean (SD) | 6.45  (1.57) | 6.46  (1.63) | 6.49  (1.63) | 6.44  (1.47) | 6.40  (1.50) | 6.71  (1.39) | 6.71  (1.38) | 6.71  (1.36) | 6.69  (1.41) | 6.68  (1.46) |
| BASFI, mean (SD) | 5.34  (2.23) | 5.48  (2.19) | 5.59  (2.11) | 5.08  (2.29) | 4.95  (2.35) | 5.28  (2.04) | 5.40  (1.96) | 5.35  (1.98) | 5.13  (2.12) | 5.12  (2.17) |
| BASMI, mean (SD) | 3.84  (1.71) | 4.10  (1.76) | 4.21  (1.77) | 3.36  (1.50) | 3.29  (1.45) | 3.11  (1.48) | 3.50  (1.54) | 3.29  (1.53) | 2.63  (1.25) | 2.69  (1.25) |
| Uveitis | 59  (21.3) | 41 (22.7) | 38  (22.9) | 18 (18.8) | 21  (18.9) | 110  (14.9) | 62 (15.4) | 80  (15.6) | 48  (14.3) | 30  (13.2) |
| IBD (Crohn’s disease/ulcerative colitis) | 18  (6.5) | 12  (6.6) | 12  (7.2) | 6  (6.3) | 6  (5.4) | 16 (2.2) | 9 (2.2) | 10 (2.0) | 7 (2.1) | 6 (2.6) |
| Psoriasis | 19  (6.9) | 11  (6.1) | 10  (6.0) | 8  (8.3) | 9  (8.1) | 45 (6.1) | 24 (6.0) | 29  (5.7) | 21 (6.3) | 16 (7.0) |
| Arthritis | 148  (53.4) | 87 (48.1) | 79  (47.6) | 61  (63.5) | 69  (62.2) | 279  (37.8) | 135  (33.5) | 178  (34.8) | 144  (42.9) | 101  (44.5) |
| Enthesitis | 100  (36.1) | 58  (32.0) | 55  (33.1) | 42  (43.8) | 45  (40.5) | 201  (27.2) | 111  (27.5) | 127  (24.8) | 90  (26.8) | 74  (32.6) |
| Dactylitis | 27  (9.7) | 13  (7.2) | 12  (7.2) | 14  (14.6) | 15  (13.5) | 47  (6.4) | 17 (4.2) | 20  (3.9) | 30 (8.9) | 27 (11.9) |

axSpA, axial spondyloarthritis; ASDAS, Ankylosing Spondylitis Disease Activity Score; BASDAI, Bath Ankylosing Spondylitis Disease Activity Index; BASFI, Bath Ankylosing Spondylitis Functional Index; BASMI, Bath Ankylosing Spondylitis Metrology Index; BMI, body mass index; CRP, C-reactive protein

**Supplementary Table S2: Inter-Reader Agreement on Sacroiliitis Eligibility (N=277)***

|  | Reader 1 | Reader 2 | Adjudicator |
| --- | --- | --- | --- |
| ML Algorithm: Kappa | 0.56 | 0.54 | 0.61 |
| ML Algorithm: Agreement (%) | 79.1 | 78.5 | 80.7 |
| ML Algorithm: ICC | 0.56 | 0.55 | 0.56 |

*This analysis excludes 5 subjects with invalid x-ray images at screening or baseline. ML: Machine learning; ICC: Intra-class correlation coefficient
